# Blockchain-Based Dynamic Consent and its Applications for Patient-Centric Research and Health Information Sharing: Protocol for an Integrative Review

**DOI:** 10.2196/50339

**Published:** 2024-02-05

**Authors:** Wendy M Charles, Mark B van der Waal, Joost Flach, Arno Bisschop, Raymond X van der Waal, Hadil Es-Sbai, Christopher J McLeod

**Affiliations:** 1 Health Administration Program Business School University of Colorado, Denver Denver, CO United States; 2 Healthcare Informatics Program University of Denver Denver, CO United States; 3 Triall Maarssen Netherlands; 4 Athena Institute Vrije Universiteit Amsterdam Amsterdam Netherlands; 5 Department of Cardiovascular Medicine Mayo Clinic Jacksonville, FL United States

**Keywords:** best practices, blockchain, clinical trial, data reuse, data sharing, dynamic consent, health care data, integrative research review, scientific rigor, technology implementation

## Abstract

**Background:**

Blockchain has been proposed as a critical technology to facilitate more patient-centric research and health information sharing. For instance, it can be applied to coordinate and document dynamic informed consent, a procedure that allows individuals to continuously review and renew their consent to the collection, use, or sharing of their private health information. Such has been suggested to facilitate ethical, compliant longitudinal research, and patient engagement. However, blockchain-based dynamic consent is a relatively new concept, and it is not yet clear how well the suggested implementations will work in practice. Efforts to critically evaluate implementations in health research contexts are limited.

**Objective:**

The objective of this protocol is to guide the identification and critical appraisal of implementations of blockchain-based dynamic consent in health research contexts, thereby facilitating the development of best practices for future research, innovation, and implementation.

**Methods:**

The protocol describes methods for an integrative review to allow evaluation of a broad range of quantitative and qualitative research designs. The PRISMA-P (Preferred Reporting Items for Systematic Review and Meta-Analysis Protocols) framework guided the review’s structure and nature of reporting findings. We developed search strategies and syntax with the help of an academic librarian. Multiple databases were selected to identify pertinent academic literature (CINAHL, Embase, Ovid MEDLINE, PubMed, Scopus, and Web of Science) and gray literature (Electronic Theses Online Service, ProQuest Dissertations and Theses, Open Access Theses and Dissertations, and Google Scholar) for a comprehensive picture of the field’s progress. Eligibility criteria were defined based on PROSPERO (International Prospective Register of Systematic Reviews) requirements and a criteria framework for technology readiness. A total of 2 reviewers will independently review and extract data, while a third reviewer will adjudicate discrepancies. Quality appraisal of articles and discussed implementations will proceed based on the validated Mixed Method Appraisal Tool, and themes will be identified through thematic data synthesis.

**Results:**

Literature searches were conducted, and after duplicates were removed, 492 articles were eligible for screening. Title and abstract screening allowed the removal of 312 articles, leaving 180 eligible articles for full-text review against inclusion criteria and confirming a sufficient body of literature for project feasibility. Results will synthesize the quality of evidence on blockchain-based dynamic consent for patient-centric research and health information sharing, covering effectiveness, efficiency, satisfaction, regulatory compliance, and methods of managing identity.

**Conclusions:**

The review will provide a comprehensive picture of the progress of emerging blockchain-based dynamic consent technologies and the rigor with which implementations are approached. Resulting insights are expected to inform best practices for future research, innovation, and implementation to benefit patient-centric research and health information sharing.

**Trial Registration:**

PROSPERO CRD42023396983; http://tinyurl.com/cn8a5x7t

**International Registered Report Identifier (IRRID):**

DERR1-10.2196/50339

## Introduction

### Rationale

Blockchain has been proposed as a critical technology to advance patient engagement and facilitate a shift toward improved patient centricity in health research [1]. For instance, in addition to being used as a means to enhance the integrity and transparency of data in clinical trials [2,3], research indicates that it can be applied to coordinate and document dynamic consent for patient-centric health information sharing [4,5] in which patients’ needs, wants, and perspectives are optimally considered [6].

Dynamic consent is a procedure that allows patients and research participants to continuously review and renew their consent to use their private health information. Compared to conventional one-time obtainment and documentation of informed consent, dynamic consent strives to give individuals greater control over their data and to ensure their preferences are respected throughout their participation [7,8]. Moreover, dynamic consent can facilitate ongoing engagement for the secondary use of data (ie, for purposes other than the initial purposes for which consent was given). Thus, dynamic consent may open up novel opportunities for ethical and compliant research, data sharing, and longitudinal researcher-patient engagement beyond the scope of the original agreement [9].

Blockchain technology offers functionality and efficiencies that are difficult to achieve with traditional data systems. First, it can be applied to facilitate dynamic consent by establishing a privacy-preserving, universally verifiable, and immutable record of the consent process [9]. The decentralized nature of blockchain ensures that these records cannot be altered by a single party, safeguarding the integrity of the participant’s documented consent independent of any 1 system or device. Moreover, blockchain offers means for mathematical and privacy-preserving verification of (consent) records and associated user identities, with no dependence on trusted intermediaries for reliable recordkeeping [10]. Such can be used for decentralized authentication and authorization, bolstering individual autonomy. In addition, using blockchain in dynamic consent can enhance the transparency of the process. Research participants can track how their data are used and can be provided with means to readily revise their consent [11]. The use of dynamic consent can inspire more people to participate in clinical research by fostering trust between participants and researchers [8]. Additionally, dynamic consent allows for continuous engagement and communication with research participants throughout and after a study, registry, or repository participation [12]. Last, blockchain technologies are currently used to automate downstream researcher access to data based on individuals’ preferences without the burden and expense of manual curation [9].

While promising, dynamic consent based on blockchain technology is a relatively new concept, and it is unclear how well it functions in real-world health research environments. Although numerous studies and pilot projects have been conducted to describe the potential application of blockchain technology to facilitate dynamic consent capabilities [13-15], it is necessary to critically evaluate the articles’ scientific methodology and results. Further, there are few best practices published about implementation methodology for blockchain-based dynamic consent. Moreover, there is a need for critical appraisal of approaches that capitalize on blockchain’s abilities to empower individuals in managing their digital data and identities. For example, self-sovereign identity (SSI) seems particularly pertinent for ongoing authentication and authorization in patient-centric research collaborations [16].

Yet, as of December 2022, the authors could not locate any published systematic or scoping review to examine the current state of blockchain-based dynamic consent features and implementations. Additionally, no reviews of blockchain-based dynamic consent are currently registered as “in progress” with PROSPERO (International Prospective Register of Systematic Reviews), the systematic review registry [17]. Therefore, there is a need for a systematic review to capture a wide range of literature to establish the scope and quality of evidence for blockchain-based dynamic consent features and implementations in clinical settings.

### Objectives

We aim to conduct a systematic integrative research review to synthesize a wide range of evidence regarding blockchain-based dynamic consent solutions, thereby informing future innovation research and practice in this domain. The goal is to identify blockchain-based dynamic consent implementations, the technology’s impact, and potential best practices for research, innovation, and implementation.

### Research Questions

The following research questions (RQs) will be used to guide the analysis and critical appraisal:

- RQ1: What are the current implementations of dynamic consent involving blockchain and their objectives for health information sharing and health research? What empirical evidence is provided for these implementations?
- RQ2: What are the risks, challenges, and opportunities of applying blockchain-based dynamic consent for health information sharing and health research?
- RQ3: What are the technical, spatial, and temporal aspects of SSI for different blockchain-based dynamic consent systems for health information sharing and health research?
- RQ4: What are the future research directions for research, innovation, and implementation of blockchain-based dynamic consent systems for health information sharing and health research?

A summary of how RQs are addressed by the various research methods is provided in Table 1.

**Table 1:** Research questions and methods to address them.

Research questions	Associated research methods
RQ1: What are the current implementations of dynamic consent involving blockchain and their objectives for health information sharing and health research? What empirical evidence is provided for these implementations?	Include only publication years since 2016 (current), and only Technology Readiness Level 6 or higher (actual implementations). Use integrative design that considers different sources and varied research methodologies. Perform systematic data extraction and evaluation on implementations’ development status and empirical evidence.
RQ2: What are the risks, challenges, and opportunities of applying blockchain-based dynamic consent for health information sharing and health research?	Perform systematic data extraction and evaluation on implementations’ effectiveness, efficiency, satisfaction, compliance, challenges, and limitations.
RQ3: What are the technical, spatial, and temporal aspects of self-sovereign identity for different blockchain-based dynamic consent systems for health information sharing and health research?	Perform systematic data extraction and evaluation on implementations’ consideration of self-sovereign identity standards and capabilities.
RQ4: What are the future research directions for designing, implementing, and validating blockchain-based dynamic consent systems for health information sharing and health research?	Systematic data extraction and evaluation of articles’ future research suggestions and description of best practices regarding design, implementation, and validation. Critical appraisal and synthesis of collected data from RQ1-4 into best practices for research and innovation.

## Methods

### Protocol and Registration

This protocol demonstrates a priori development of the research plan. It was registered at PROSPERO on February 15, 2023 (registration number CRD42023396983) [18], before initiating literature review activities. In addition, a timestamped and immutable cryptographic record of the protocol was generated using blockchain-anchoring technology by Triall (Clinblocks BV), allowing for indisputable and independent verification of the protocol and its exact contents at the registered time.

The PRISMA-P (Preferred Reporting Items for Systematic Reviews and Meta-Analyses Protocols) checklist [19,20] was referenced to prepare this protocol.

### Study Design

The review will accumulate and synthesize the global published literature about blockchain-based dynamic consent, including quantitative, qualitative, and mixed methods research articles. We will use integrative research review methodology for a comprehensive review of diverse literature [21]. Specifically, integrative reviews aim to provide a comprehensive and holistic understanding of a particular phenomenon or problem by synthesizing evidence from diverse research methodologies. For example, an integrative review may include case studies, observational studies, mixed methods, and qualitative methods that inform theory. By synthesizing a combination of diverse research methodologies, an integrative review allows researchers to develop new theories or models as well as identify gaps in the literature that need to be addressed by future research [22,23]. We plan to identify literature gaps and suggest methods for strengthening future research designs accordingly [24]. By critically appraising current implementations of blockchain-based dynamic consent, we also plan to inform best practices for future innovation and implementation.

To ensure precision, we will adopt the following definitions:

A blockchain is a decentralized, distributed ledger that records information about transactions or activities across a network of computers. Blocks consist of interconnected, encrypted groups of records [25].

Dynamic consent is a method of electronic consent that is flexible, configurable, and can honor an individual’s consent preferences across a spectrum of choices over time [7,8].

The protocol is organized to use the integrative review methodology proposed by Whittemore and Knafl [21] and augmented by the PRISMA-P checklist [19,20].

### Problem Identification

To progress with blockchain-based dynamic consent, it is critical to advance high-quality research and establish guidance for innovation and implementation. However, most papers to date have concentrated on theoretical applications and proofs of concept. Despite the acknowledged requirement for high-quality data and scientific rigor [26], evidence collection has received little attention.

### Literature Search

The integrative review will focus on published articles about blockchain-based dynamic consent. Databases selected to search the indexed peer-reviewed academic literature include CINAHL, Embase, Ovid MEDLINE, PubMed, Scopus, and Web of Science (all databases). Gray literature (which is described in various ways but typically includes nearly everything not published in a peer-reviewed journal [27]) will be included in the literature review because a significant portion of relevant research and innovation in this fast-moving field takes place outside of academia. Further, gray literature allows for a more comprehensive assessment of the field’s progress [28]. While gray literature can be captured in some of the indices listed above, additional search engines selected to identify gray literature include Electronic Theses Online Service, ProQuest Dissertations and Theses, Open Access Theses and Dissertations, and Google Scholar.

### Eligibility Criteria

Eligibility criteria reflect PROSPERO questions and requirements [29]. Not all PROSPERO data fields are pertinent for this integrative review. The eligibility criteria are summarized and presented in Textbox 1.

**Article inclusion and exclusion eligibility criteria.**

**Inclusion criteria**

- Original research articles (architecture, system designs, framework, scheme, model, platform, approach, protocols, test results, and algorithms)
- Any type of research methodology, including quantitative, qualitative, mixed methods, and descriptive narratives
- Describes an actual blockchain-based dynamic consent system (technology readiness level 6 or higher)
- Solutions are implemented in, or intended for, human health-oriented care or research contexts
- Publication years since 2016
- English language articles

**Additional sources of gray literature for inclusion criteria**

- Scientific or government reports
- Books and book chapters
- Conference papers or proceedings
- Theses

**Exclusion criteria**

- Review articles that summarize a body of existing literature (although review articles will be examined to perform backward literature tracking)
- Abstracts only
- Letters to the Editor
- Non-English articles
- Secondary research where an author describes work from another publication
- Articles discussing only proposed, potential, or theoretical applications of blockchain-based dynamic consent
- Articles unrelated to the topic

**Additional sources of gray literature for exclusion criteria**

- Magazine publications
- Interviews
- White papers
- Patents
- Preprints self-posted by the author

#### Types of Articles to Be Included

Inclusion and exclusion criteria reflect academic and gray literature sources to capture a vast body of literature for the integrative review. These criteria are deliberately broad because few empirical studies are available for inclusion. Therefore, we will include quantitative, qualitative, mixed methods, and descriptive articles from various sources. Further, review articles are included to permit backward citation tracking. A population, intervention, control, and outcomes format was used to guide the article selection criteria as follows.

#### Participants and Population

Articles must feature participants who would use a blockchain-based dynamic consent solution. Populations may include—but are not limited to—patients, research participants, providers, staff members, and research administrators. Some studies may not assess the actual users.

#### Interventions and Exposures

Rather than a health intervention, participant exposure involves interaction with a dynamic consent solution. The blockchain-based dynamic consent solution must demonstrate sufficient development progress for evaluation. We evaluated the technology described in each article using a modified Technology Readiness Level (TRL) framework that includes technology descriptions from both the US Government Accountability Office [30] and the US Department of Defense [31]. TRL frameworks provide guidelines about the nature of evidence and progress expected for each level of technology development on a scale of 1 (idea formulation and review of scientific literature) to 9 (ready for full-scale production and commercialization). Government Accountability Office guidelines allow consideration and early negotiation with vendors whose products meet the criteria of TRL 6 (representative model or prototype in a relevant environment) or higher. Therefore, for this integrative review, descriptions of dynamic consent solutions must meet the criteria for TRL 6 or higher for inclusion. Our preliminary examination of the full-text articles has confirmed a sufficient body of literature with blockchain-based dynamic consent products at and above this threshold.

#### Comparators and Control

While we would like to capture comparisons of blockchain-based systems to other comparator systems, we are unaware of any published head-to-head comparisons. Therefore, we included studies without comparators if they meet all other eligibility criteria.

#### Context

The studies must apply to a health research context, which is, they must describe intended or actual application to the sharing and use of health information for research purposes. Publication years must be 2016 or later to capture the modern implementations of blockchain in this context.

#### Main Outcome

The review is intended to capture evidence of progress with blockchain, and dynamic consent technology related to health research. Several components will be collected about the implementation or commercialization stage, effectiveness, efficiency, satisfaction, regulatory compliance, and methods of managing identity.

### Search Terms

The search strategies were determined through team discussion and were reviewed by an experienced academic librarian at the University of Colorado Denver Auraria Library.

The blockchain-based dynamic consent search strategy includes 2 primary blocks of search terms: “blockchain” and “dynamic consent” within a health care or health research setting. The blocks of terms include synonyms and related concepts, such as using the search term “distributed ledger” for “blockchain.”

Because exact terms, such as “dynamic consent,” may not be used in the desired articles, a list of synonyms was generated for “dynamic,” including: “progressive,” “personalized,” “customized,” “interactive,” “modify,” “modifiable,” “revocation,” “revocable,” and “revoking.” These synonyms will be searched within the proximity of other pertinent terms. For example, we propose using synonyms of “dynamic” within 5 words of terms representing “consent” as a verb or noun, including: “consent,” “permission,” “grant,” “authorize,” “authorization,” “allow,” “agree,” and “agreement.”

Search strategies used character substitutions, such as “decentrali?ed,” to capture American and British English spelling variations (eg, decentralized and decentralised, respectively). Further, word truncations were used to capture related word endings, such as “consent*,” to capture “consent,” “consents,” and “consenting.” The search strategy was customized for each index or database’s unique syntax and capabilities. The complete search terms and syntax are presented for a sample MEDLINE search in Multimedia Appendix 1.

Additional scientific articles were identified using manual backward and forward citation tracking of review articles obtained during the search. Backward citation tracking, also referred to as “backward chaining,” “footnote chasing,” and “reference list searching,” is an umbrella term for finding articles directly or indirectly from the reference section of articles being reviewed [28]. Forward citation tracking, also called “forward chaining,” aims to identify additional literature among the articles that cite one of the selected articles [32]. These additional abstracts were obtained and reviewed for inclusion.

Several gray literature articles were incidentally identified using the search process described above. Theses and dissertations were identified with iterations of the search terms to augment the gray literature search process. Google Scholar was searched using the advanced search screen and Boolean operators. Forward citation tracking was facilitated by clicking the “cited by” option.

### Article Selection and Screening

Search results were imported and deduplicated using Covidence Systematic Review Management software (Veritas Health Innovation Ltd). A total of 2 reviewers (WMC and MBW) independently reviewed titles and abstracts (and keywords, when applicable) to determine potential eligibility for inclusion in the review. Any abstracts considered too ambiguous or where reviewers disagreed were resolved by a third reviewer (JF) or by examining the full text.

To facilitate full-text review and abstraction, Covidence automatically imported the open-access articles, and the remaining articles were manually imported. The articles selected for abstraction were also imported into EndNote (version X9; Clarivate) citation management software to facilitate manuscript preparation.

### Full-Text Review

A total of 2 reviewers (WMC and MBW) will independently review the publications to verify that eligibility criteria are met. While the articles do not specify a TRL level, the reviewers will attempt to discern whether the blockchain-based dynamic consent technologies described in the articles meet the criteria for TRL 6 or higher. Any reviewer disagreement will be resolved by a third reviewer (JF) or by discussions between reviewers.

### Data Extraction and Evaluation

Although integrative reviews do not typically include a quality appraisal, we have elected to use the validated Mixed Method Appraisal Tool (MMAT) [33] to capture data on the studies’ quality. This aligns with established guidelines for organizing and synthesizing a wide range of literature [22,23]. Moreover, we recognize that there is an increased risk of bias in articles where the authors describe their own products with the goal of future commercialization. The MMAT is brief (only 5 yes or no questions per article) and is designed to assess the methodological strength of studies with diverse designs, consistent with an integrative review. Questions pertain to methodological quality, interpretations, and risks of bias. This tool has been assessed and updated for reliability [34] and content validity [35]. A total of 2 reviewers (WMC and MBW) will independently address the MMAT quality appraisal questions of the eligible literature and will extract all pertinent information from each article. A third reviewer (JF) will integrate the reviews from the 2 reviewers or request collaborative discussions among all reviewers.

The remaining data extraction questions were developed by WMC and MBW and refined by the other team members. Questions were programed into Covidence and tracked with an audit trail. Key information planned for extraction includes: (1) article characteristics: author or authors, year of publication, title, and journal; (2) context and setting for actual or intended blockchain-based dynamic consent implementation: country of intended implementation, setting of implementations (eg, hospital, clinic, or research organization), and intended users; (3) details about blockchain-based dynamic consent solution: name given to the technology (if any), stated purposes or objectives of the product, blockchain platform used (if specified), capabilities of integrations with other data sources, maturity of the technology, stated compliance with regulations, SSI capabilities; (4) evidence about the blockchain-based dynamic consent solution: comparisons with other technologies, evaluation with patients or research participants, nature, and scope of evidence presented; and (5) additional questions about scientific integrity and future research: descriptions of challenges and limitations, whether evidence is presented objectively, descriptions of best practices, and recommendations for future research.

### Data Synthesis

Categorical data will be summarized by the percentage of articles or blockchain-based dynamic consent products by category.

Information pertaining to the research questions will be organized using qualitative summary narratives—comparing and contrasting blockchain-based dynamic consent approaches [36]. If necessary, a coding manual will be developed to increase the consistency of coding categorization and the synthesis of subthemes [37,38]. The interpretations will consider article quality, representativeness, and bias or objectivity [36].

## Results

### Article Selection and Screening

Using the search strategies and databases described above, 637 articles were identified. A total of 145 duplicates were removed, resulting in 492 articles eligible for preliminary screening. Title and abstract screening removed 312 articles. A total of 180 articles are available for full-text review against inclusion criteria, confirming a sufficient body of literature for project feasibility.

### Presentation

The review will present data as a narrative, supported by tables and graphs to display the characteristics of included articles and implementations of blockchain-based dynamic consent. The structure of the narrative synthesis will reflect the objective and research questions. The flow of information through the review phases will be generated by Covidence and displayed as a PRISMA (Preferred Reporting Items for Systematic Reviews and Meta-Analyses) 2020 flow diagram [39].

## Discussion

### Key Findings

While the results will not be known until we complete the integrative review, we plan to interpret the findings and their implications in the context of the 4 research questions. In discussing findings and implications, we will refer to the subthemes synthesized by evaluating data extracted from the articles.

### Potential Impact of the Review

Individual patients and research participants are increasingly given more rights to control the uses of their data through privacy regulations [40-42] and health information interoperability requirements [43,44]. In addition, patient-centric research initiatives are considered by industry, academia, and regulatory stakeholders globally as a promising means to overcome clinical research and development delays, inefficiencies, and high failure rates [45]. Therefore, technologies that facilitate patient-centricity, such as blockchain-based dynamic consent, are increasingly relevant for health care and health research organizations. It underscores the importance of rigorously reviewing these technologies and their impact, elevating scientific rigor, and establishing best practices for research, innovation, and implementation.

#### Elucidating Technology Benefits

Extant studies that suggest blockchain-based technologies for health care settings typically involve prototypes, proof-of-concept technologies, or minimum viable products, where nearly all purported results are theoretical [46]. This protocol and subsequent integrative review publication highlight the relevance and methods for evaluating technology readiness and empirical evidence. Similarly, some studies promote blockchain products and possible benefits but do not explain how products are designed to address specific problems. For example, Durneva et al [47] performed a systematic review of blockchain-based systems for health care and examined how well-proposed solutions aligned with organizational goals. In their review, the authors noted that only 10% (7/70) of products were designed to reduce inefficiencies. As organizations publish more literature about these technology developments, this protocol provides methodology that facilitates critical appraisal of studies and presented evidence.

With the recognition that it is necessary to manage users’ identities for a digital consent solution used over time [48]—and possibly across multiple devices—we believe this literature review will also shed light on the identity management features that blockchain could underpin.

#### Elevating Scientific Rigor

This work can inform and improve future research designs on blockchain-based dynamic consent. In 2019, Treiblmaier [26] noted that the quality of blockchain research was lagging, and he offered guidelines for designing and publishing case studies to improve evidence quality. Years later, authors still emphasize that blockchain research must have more scientific rigor for the work to be respected [49-51]. When addressing blockchain-based dynamic consent literature quality, we will offer deeper insight into gaps, weaknesses, and ways to address these.

#### Offering Best Practices

This integrative review is expected to identify optimal approaches to implementing and evaluating the effectiveness of blockchain-based dynamic consent technologies. While blockchain products are being developed for health research environments, few implementation models exist [52]. Additionally, few articles describe their products’ limitations or weaknesses, contributing to narrow and unrealistic perspectives [53]. Based on the data, we plan to discuss how blockchain-based dynamic consent solutions must be designed to address specific issues in health information sharing and health research. This review will aggregate all information from the articles about strengths and weaknesses—plus draw from our real-world experience—to offer best practices for blockchain technology implementation in this domain. To enhance understanding and practical relevance, we intend to discuss these best practices in an exemplary implementation case, for example, blockchain-based dynamic consent for collecting and exchanging health information and managing informed consent across research centers in the context of longitudinal cardiovascular research projects with extensive and diverse participant cohorts.

### Limitations

This integrative research review has several limitations. First, we acknowledge that this is a relatively new area of research and innovation. As a result, the literature base is still relatively small and may not facilitate a comprehensive understanding of the technology and its potential applications. Another limitation is that many published studies on blockchain-based dynamic consent describe early stages of development rather than being based on real-world implementations [14]. This means that they may not reflect the challenges and limitations encountered in practical applications of the technology. Last, some published studies on blockchain-based dynamic consent may be based on small-scale or pilot projects, which may not represent how the technology would perform in a larger, more complex health research setting [47]. To mitigate these limitations, we have conducted a preliminary full-text examination of eligible articles to confirm a sufficient body of literature on implementations at a sufficiently progressed technology readiness level (ie, TRL 6 or higher). The quality of studies and their focal implementations will be appraised systematically using the MMAT. Besides, the early stages of this research arguably increase the relevance of critically evaluating current approaches and synthesizing best practices for future research, innovation, and implementation.

### Conclusions

The review will provide a comprehensive picture of the progress of emerging blockchain-based dynamic consent technologies and the rigor with which implementations are approached. Resulting insights are expected to inform best practices for future research, innovation, and implementation to benefit patient-centric research and health information sharing.

## Appendix

Multimedia Appendix 1 PubMed Search Syntax.

## Abbreviations

MMAT: Mixed Method Appraisal Tool
PRISMA: Preferred Reporting Items for Systematic Reviews and Meta-Analyses
PRISMA-P: Preferred Reporting Items for Systematic Review and Meta-Analysis Protocols
PROSPERO: International Prospective Register of Systematic Reviews
RQ: research question
SSI: self-sovereign identity
TRL: Technology Readiness Level

## Data Availability

Data sharing is not applicable to this article as no data sets were generated or analyzed during this study.
