# Supplementary material for: Blockchain-Based Dynamic Consent and its Applications for Patient-Centric Research and Health Information Sharing: Protocol for an Integrative Review
Source: JMIR Res Protoc. 2024 Feb 5;13:e50339. doi: 10.2196/50339 (PMC10877491; doi:10.2196/50339)
Supplement: Multimedia Appendix 1 [file resprot_v13i1e50339_app1.docx]

## Multimedia Appendix

## PubMed Search Syntax

#1 blockchain[tiab] OR blockchain[ot]

#2 "block chain"[tiab] OR “block chain”[ot]

#3 “distributed ledger”[tiab:~3] OR “distributed ledger”[ot]

#4 “decentralized ledger”[tiab:~3] OR “decentralized ledger”[ot]

#5 “decentralised ledger”[tiab:~3] OR “decentralised ledger”[ot]

#6 “decentralized identity”[tiab:~3] OR “decentralized identity”[ot]

#7 “decentralised identity”[tiab:~3] OR “decentralised identity”[ot]

#8 “decentralized identifier”[tiab:~3] OR “decentralized identifier”[ot]

#9 “decentralised identifier”[tiab:~3] OR “decentralised identifier”[ot]

#10 web3[tiab] OR web3[ot]

#11 #1 OR #2 OR #3 OR #4 OR #5 OR #6 OR #7 OR #8 OR #9 OR #10

#12 dynamic* [tiab] OR progressive* [tiab] OR personali* [tiab] OR customi* [tiab] OR interactive [tiab] OR grant* [tiab] OR modif* [tiab] OR revoca* [tiab] OR revok* [tiab]

#13 dynamic[ot] OR progressive* [ot] OR personali* [ot] OR customi* [ot] OR interactive [ot] OR grant* [ot] OR modif* [ot] OR revoca* [ot] OR revok* [ot]

#14 #12 OR #13

#15 consent, informed [mh]

#16 consent[tiab] OR permission[tiab] OR share[tiab] OR sharing[tiab] OR authoriz*[tiab] OR authoris*[tiab] OR allow*[tiab] OR agree*[tiab]

#17 consent[ot] OR permission[ot] OR share[ot] OR sharing[ot] OR authoriz*[ot] OR authoris*[ot] OR allow*[ot] OR agree*[ot]

#18 #15 OR #16 OR #17

#19 #14 AND #18

#20 #11 AND #19

#21 Remove articles that are abstracts only and articles published before 2016.
